# The Role of SOX2 and SOX9 Transcription Factors in the Reactivation-Related Functional Properties of NT2/D1-Derived Astrocytes

**DOI:** 10.3390/biomedicines12040796

**Published:** 2024-04-03

**Authors:** Vanda Balint, Mina Peric, Sanja Dacic, Danijela Stanisavljevic Ninkovic, Jelena Marjanovic, Jelena Popovic, Milena Stevanovic, Andrijana Lazic

**Affiliations:** 1 Laboratory for Human Molecular Genetics, Institute of Molecular Genetics and Genetic Engineering, University of Belgrade, Vojvode Stepe 444a, 11042 Belgrade, Serbia; vanda@imgge.bg.ac.rs (V.B.); minaperic@imgge.bg.ac.rs (M.P.); danijelastanisavljevic@imgge.bg.ac.rs (D.S.N.); jxm2322@miami.edu (J.M.); jelena.popovic@northwestern.edu (J.P.); milenastevanovic@imgge.bg.ac.rs (M.S.); 2 Institute of Physiology and Biochemistry “Ivan Djaja”, Faculty of Biology, University of Belgrade, Studentski trg 16, 11158 Belgrade, Serbia; sanjas@bio.bg.ac.rs; 3 Serbian Academy of Sciences and Arts, Kneza Mihaila 35, 11001 Belgrade, Serbia

**Keywords:** reactive astrocytes, *SOX* genes, NT2/D1 cell line, glutamate uptake, brain tissue repair, astrogliosis

## Abstract

Astrocytes are the main homeostatic cells in the central nervous system, with the unique ability to transform from quiescent into a reactive state in response to pathological conditions by reacquiring some precursor properties. This process is known as reactive astrogliosis, a compensatory response that mediates tissue damage and recovery. Although it is well known that SOX transcription factors drive the expression of phenotype-specific genetic programs during neurodevelopment, their roles in mature astrocytes have not been studied extensively. We focused on the transcription factors SOX2 and SOX9, shown to be re-expressed in reactive astrocytes, in order to study the reactivation-related functional properties of astrocytes mediated by those proteins. We performed an initial screening of SOX2 and SOX9 expression after sensorimotor cortex ablation injury in rats and conducted gain-of-function studies in vitro using astrocytes derived from the human NT2/D1 cell line. Our results revealed the direct involvement of SOX2 in the reacquisition of proliferation in mature NT2/D1-derived astrocytes, while SOX9 overexpression increased migratory potential and glutamate uptake in these cells. Our results imply that modulation of *SOX* gene expression may change the functional properties of astrocytes, which holds promise for the discovery of potential therapeutic targets in the development of novel strategies for tissue regeneration and recovery.

## 1. Introduction

Astrocytes are the main homeostatic cells in the central nervous system (CNS), with essential roles in both physiological and pathological conditions [1,2]. In the healthy CNS, these cells exert many important functions, such as neuronal transmission and synaptic plasticity [3], regulation of blood flow, homeostasis of extracellular fluids, metabolic supply, and brain antioxidant defense [2,4]. Astrocytes are also responsible for the uptake and recycling of glutamate, a major excitatory neurotransmitter in the CNS [2], through glutamate transporter-1 (GLT1)/excitatory amino acid transporter 2 (EAAT2) and glutamate and aspartate transporter (GLAST)/excitatory amino acid transporter 1 (EAAT1) [5,6,7]. In the adult brain, astrocytes have the unique ability to resume proliferation and reacquire the properties of glial precursors present in the stages of early development [8,9,10]. This phenomenon, known as reactive astrogliosis, is induced in response to pathological conditions in the brain, including mechanical trauma, ischemia, infections, and different neurodegenerative diseases [4,11,12]. This is a complex, evolutionarily conserved process that serves as a compensatory response and can mediate tissue damage and recovery [12]. Reactive astrocytes can exert both beneficial and detrimental effects on neural tissue repair through the loss of their physiological functions or the gain of detrimental functions [13,14]. Reactive astrocytes go through vast morphological, transcriptional, and functional changes. The most prominent changes in reactive astrocytes include cell body hypertrophy, increased proliferation, up-regulation of glial fibrillary acidic protein (GFAP) [1,15,16], as well as re-expression of the progenitor markers vimentin and nestin [15,17,18]. In cases of severe brain injury, reactive astrocytes migrate towards the site of injury, where they proliferate and form a glial scar [19] that prevents the spread of inflammation but also inhibits axonal regeneration and synapse formation [20]. Also, the down-regulation of glutamate transporters in reactive astrocytes has been reported [21,22], which has a detrimental effect on surrounding neural tissue through glutamate-mediated excitotoxicity. Even though reactive astrocytes and glial precursors share some phenotypic characteristics, it is not completely understood whether they are governed by the same molecular mechanisms.

The latest studies imply that targeting the functional properties of reactive astrocytes may switch these cells to become a part of potent endogenous defense mechanisms that could be manipulated to promote neuronal survival and recovery [1,12]. Therapeutic strategies based on the functional modulation of reactive astrocytes, including viral-mediated gene therapy, are aimed at enhancing their beneficial roles while minimizing or completely eliminating undesired outcomes [23,24]. Accordingly, the elucidation of the molecular mechanism underlying reactivation-related functional properties of astrocytes is of great importance for developing new neuroprotective strategies.

Limited access to human astroglia for pre-clinical studies has been a major obstacle delaying our understanding of their role in the brain under physiological and pathological conditions [25]. Since the availability of human primary astrocytes is limited, astrocytes deriving from human pluripotent embryonal teratocarcinoma NT2/D1 cells are good alternatives for in vitro studies. This cell line is able to differentiate along the neural lineage when treated with retinoic acid, yielding both neurons and astrocytes [26,27]. NT2/D1-derived astrocytes (NT2/A) represent an adequate model system for studying human astrocytes as they have similar properties to primary astrocytes, including morphology, the presence of intermediate filament proteins, an active glutamate transport system, and growth arrest during the maturation process [26,27]. Moreover, NT2/A exert a reactive response to the injury, as evidenced by the rapid change in morphology, increased incorporation of nuclear Bromodeoxyuridine (BrdU), and intensive GFAP staining [27].

Sry-related HMG box (SOX) transcription factors are well-known regulators of cell fate decisions during development [28]. SOX2 and SOX9 are involved in the maintenance of neural stem cell populations in both the embryonic and adult CNS [29,30,31,32]. SOX2 promotes neural proliferation, survival, and self-renewal [30,32]. In the adult mouse brain, SOX2 is expressed in neurogenic regions and early postnatal proliferative astrocytes [32,33]. SOX9 plays an important role in a transcriptional regulatory cascade that operates during the gliogenic switch, and it is crucial for the onset of gliogenesis [34,35]. Several studies have shown that both SOX2 and SOX9 become re-expressed in reactive astrocytes [33,36,37], but the molecular events triggered by these transcription factors are not yet completely understood.

The aim of our study was to identify the reactivation-related functional properties of astrocytes governed by SOX2 and SOX9 transcription factors. Special attention has been given to SOX9-governed mechanisms since the latest findings have shown that overexpression of SOX9 alone is sufficient to induce the formation of functional astrocytes from human induced pluripotent stem cells (hiPSCs) [25,38]. These data emphasize the importance of this transcription factor in regulating astrocyte physiological functions. The initial screening of SOX2 and SOX9 expression after injury was performed ex vivo in rats after sensorimotor cortex ablation (SCA), and further gain-of-function studies were performed in vitro using NT2/A. Our initial screening of SOX2 and SOX9 expression after brain injury in the rat cortex showed up-regulation of these transcription factors in reactive astrocytes around the lesion area. Our functional studies showed that SOX2 overexpression leads to an increase in NT2/A viability and proliferation. Further, SOX9 overexpression promoted migration and glutamate uptake in NT2/A. We also demonstrated that SOX9 re-activates the same transcriptional regulatory network in mature NT2/A that is also active during gliogenesis, as shown by increased expression of the nuclear factor 1 A-type (*NFIA*) and APC Down-Regulated 1 (*APCDD1*) genes, as well as the expression of solute carrier family 1 member 3 (*SLC1A3*) gene coding for the GLAST/EAAT1 protein.

Accordingly, our results revealed that transcription factors SOX2 and SOX9 govern important reactivation-related functional features of astrocytes. Although more comprehensive studies are needed to understand the exact roles of these transcription factors in the complexity of reactive astrogliosis, here we made a step forward, showing that modulation of *SOX2* and *SOX9* gene expression may alter the functional properties of astrocytes. Our data hold promise for the development of novel potential therapeutic strategies for neural tissue regeneration and recovery.

## 2. Materials and Methods

Animals Male Wistar rats, 10 weeks old, were kept under standard conditions (12 h of light/darkness, constant ambient temperature of 23 ± 2 °C, and 50–60% relative humidity) with ad libitum access to food and water. The experimental procedures were approved by the Ethics Committee of the University of Belgrade (Application No. 61206-2915/2-20) and conducted in strict accordance with EU Directive 2010/63/EU on the protection of animals used for scientific purposes.

Experimental procedure. Animals were randomly divided into two groups: animals with suction-induced sensorimotor cortex ablation (SCA group) and age-matched intact animals (control group). The details of the surgical procedure used for this experiment have been described previously [39]. Briefly, rats were anesthetized with an intraperitoneal injection of Zoletil^®^50 (50 mg/kg; Virbac, Carros, France) and placed in a stereotaxic frame. The scalp was shaved and cut along the midline with a scalpel. The coordinates of the craniotomy were 2 mm anterior to the bregma, 4 mm posterior to the bregma, and 4 mm lateral to the midline. The right sensorimotor cortex, which lays under the craniotomy site, was carefully aspirated through a polypropylene tip, leaving the white matter layer intact. After returning the dura and displaced bone, the skin was sutured, and the animals were kept warm until awakening. The animals of both groups, the SCA group and the age-matched control group, were sacrificed 10 days after the injury.

Cell culture and differentiation. Human pluripotent NT2/D1 embryonal carcinoma cells (ATCC^®^ CRL-1973™, Manassas, VA, USA) were maintained as previously described [40]. The differentiation of NT2/D1 cells in culture was induced by adding 10 μM all-*trans* retinoic acid (Sigma-Aldrich, St. Louis, MI, USA) for four weeks. Neuronal cells were removed from underlying adherent cells by mechanical shaking according to the published protocol [41]. NT2/D1-derived astrocytes (NT2/A) were obtained from the remaining adherent cell population, as previously described [27]. Briefly, after the removal of NT2/D1-derived neurons (NT2/N), the remaining cells were trypsinized and replated in new T75 flasks. NT2/A were maintained for an additional five weeks in a growth medium containing high-glucose DMEM (52100039, Gibco, Paisley, Scotland, UK) with 10% fetal bovine serum (FBS) (10270-106, Gibco) and sub-cultured when confluent. NT2/A were maintained at 37 °C with 10% CO_2_ and 95% humidity. 

Lentiviral transduction of NT2/A. Lentiviral particles were produced in HEK-293T cells (ATCC^®^ CRL-1573™) by transient cotransfection using PEI MAX™ transfection reagent (24765-100, Polysciences Inc., Warrington, PA, USA) according to the manufacturer’s instructions. To achieve SOX9 overexpression in 4-week-old NT2/A, we used lentiviral particles obtained from HEK cells upon their cotransfection in a 10 cm culture dish using 10 μg of either lentiviral pwpXL-SOX9 transduction construct (36979, Addgene, Watertown, MA, USA) or pwpXL-empty vector control (12257, Addgene), as previously described [42]. To achieve SOX2 overexpression in 4-week-old NT2/A, we used lentiviral particles obtained from HEK cells after their cotransfection in a 10 cm culture dish using 10 µg of either lentiviral pSin-SOX2 transduction construct or pSin-empty vector as previously described [43]. Lentiviral particles were harvested 48 h after transfection of HEK-293T cells. Additionally, 4-week-old NT2/A were incubated in the medium containing lentiviral particles and 8 μg/mL Polybrene (TR-1003, Sigma-Aldrich) overnight. Puromycin selection started 48 h after transduction. For the next three days, cells were maintained in a growth medium with the addition of 0.1 μg/mL puromycin (selection medium). To test the puromycin efficiency, non-transduced cells were grown in the selection medium. The cultured puromycin-resistant cells were used for further experimental analysis. 

Cell viability assay. The viability of NT2/A after SOX2 and SOX9 transduction was tested using the CellTiter 96^®^ Aqueous One Solution Cell Proliferation Assay (MTS assay, G3582, Promega, Madison, WI, USA). Five days after transduction with SOX2 and SOX9 lentiviral constructs and their corresponding control vectors, cells were seeded in 96-well plates at a density of 1 × 10^4^ cells per well. The assays were performed according to the manufacturer’s instructions. Colorimetric measurements were performed with an Infinite 200 PRO microplate reader (Tecan, Männedorf, Switzerland). The assays were conducted in six technical replicates and repeated in three independent experiments.

Scratch wound healing assay. The potential of cells to migrate was investigated by a wound healing assay (a wound scratch assay) as previously described [44]. Two-week-old NT2/A were seeded in 10 cm culture dishes (4 × 10^5^ cells per dish) and cultured until the fourth week of maturation, when they reached confluence (5 × 10^5^ cells per dish). The confluent cell monolayer was scratched with a 200 µL tip. Cells were washed once with phosphate-buffered saline (PBS) to remove detached cells and then incubated in a fresh growth medium. Cell migration was monitored with the DM IL LED Inverted Microscope (Leica Microsystems, Wetzlar, Germany). The migratory potential of SOX9-overexpressing cells was analyzed 24 h after the initial scratch. Analysis of the migration of SOX2-overexpressing cells was conducted 36 h after the initial scratch due to their slower migration rate. The Leica Microsystems LAS AF-TCS SP8 software v4.12 was used to analyze wound closure images. For the analysis of cell migration, three independent experiments were performed. 

Forskolin treatment and in vitro injury. Astrocyte reactivation was induced by chemical treatment (forskolin) or mechanically by scratch injury. Four-week-old NT2/A plated on geltrex^®^-coated (A1413302, Gibco) glass coverslips were treated with 10 μM forskolin (F3917, Sigma-Aldrich) in growth medium for 24 h. Cells were then fixed with 4% paraformaldehyde (PFA) and further used for immunocytochemical experiments. To analyze the expression of SOX2 and SOX9 in NT2/A reactivated by scratch injury, two-week-old NT2/As were seeded on 10 cm cell culture dishes (4 × 10^5^ cells per dish) and cultured until the fourth week of maturation, when they reached confluence (5 × 10^5^ cells per dish). The confluent cell monolayer was scratched with a 200 µL tip. The cells were collected at different time points (0, 8, 24, and 36 h) after injury. Whole-cell lysates were isolated from cell pellets and used for Western blot experiments.

Reverse transcription (RT)-PCR analysis. Total RNA was isolated, treated with DNase I, and used for cDNA synthesis as previously described [45]. The cDNA was used as a template for the amplification of *SOX9*, *SOX2*, *NFIA*, *APCDD1*, and *SLC1A3* using Power SYBR Green PCR Master Mix (A25742, Applied Biosystems, Waltham, MA, USA) according to the manufacturer’s protocols. The experiment was conducted with at least three biological replicates. Amplification was performed in a 7500 Real-Time PCR System (Applied Biosystems) and CFX Opus Real-Time PCR Systems (Biorad, Hercules, CA, USA). All reactions were run in triplicate, and the mean value was calculated. The relative expression levels of these genes were calculated using the ΔΔCt method and normalized to *GAPDH* as an endogenous control. The sequences of primers used in this study are: *SOX9*: 5′-CTTCTGAACGAGAGCGAGA-3′ (forward) and 5′-CTGCCCGTTCTTCACCGACTTC-3′ (reverse), *NFIA*: 5′-TAATCCAGGGCTCTGTGTCC-3′ (forward) and 5′-CCTGCAGCTATTGGTGTCTG-3′ (reverse), *APCDD1*: 5′-AAGGAGTCACAGTGCCATCA-3′ (forward) and 5′-GCCTGACCTTACTTCACAGCC-3′ (reverse), *SOX2*: 5′-CCCCTGGCATGGCTCTTGGC-3′ (forward) and 5′-TCGGCGCCGGGGAGATACAT-3′ (reverse), *SLC1A3*: 5′-GGTTGCTGCAAGCACTCATCAC-3′ (forward) and 5′-CACGCCATTGTTCTCTTCCAGG-3′ (reverse), and *GAPDH*: 5′-GCCTCAAGATCATCAGCAATGC-3′ (forward) and 5′-CCACGATACCAAAGTTGTCATGG-3′ (reverse).

Western blot. Whole-cell lysates were prepared from NT2/A as previously described [46]. Briefly, confluent monolayers of cells from 10 cm cell culture dishes were trypsinized, centrifuged (1300 rpm for 5 min), and pellets (5 × 10^5^ cells for mature 3-week-old and 4-week-old NT2/A and 1.5 × 10^6^ cells for 1-week-old and 2-week-old NT2/A) were resuspended in TD lysis buffer ((1% Triton X-100, 50 Mm Tris-HCL (pH 7.5), 250 Mm NaCl, 5 mM EDTA, and protease inhibitor cocktail (Roche diagnostics GmbH, Hellas, SA, USA), shaken for 15 min on ice, and then centrifuged (13,000× *g*, 4 °C, 15 min). Obtained lysates were quantified by Bradford protein assay (39222.02, Serva Electrophoresis GmbH, Heidelberg, Germany). The same amount of proteins (30 μg) per sample were separated by SDS-PAGE on a 12% resolving gel and then electrotransferred to the Immobilon-P Transfer Membrane (Millipore, Burlington, MA, USA).

After blocking with 5% non-fat milk/Tris-buffered saline with 0.1% Tween^®^ 20 detergent (TBST) for 1 h at room temperature (RT), membranes were incubated at 4 °C overnight with the following primary antibodies: rabbit anti-SOX2, in 5% bovine serum albumin (BSA)/TBST (1:3000; 39823, Active Motif, Carlsbad, CA, USA); rabbit anti-SOX9 in 5% BSA/TBST (1:500; ab5535, Merck, Rahway, NJ, USA); rabbit anti-GFAP in 5% non-fat milk/TBST (1:1000; Z0334, Dako, Glostrup, Denmark); mouse anti-tubulin in 5% non-fat milk/TBST (1:100.000; ab56676, Abcam, Waltham, MA, USA); and mouse anti-GAPDH in 5% non-fat milk/TBST (1:15.000; NBP2-27103SS, Novus Biologicals, Centennial, CO, USA). The membranes were incubated for 1 h at RT with the horseradish peroxidase-conjugated anti-rabbit antibody (A16023, ThermoFisher Scientific, Waltham, MA, USA) and horseradish peroxidase-conjugated anti-mouse secondary antibody (1:10,000; 15014, Active Motif) in 5% non-fat milk/TBST. Immunoreactive bands were detected by chemiluminescence (WBKLS0050, Immobilion substrate, Millipore, Burlington, MA, USA) using either X-ray films or the Biorad ChemiDoc Imaging system. Molecular weight markers used in experiments were: Chameleon^®^ Duo Pre-stained Protein Ladder (928-6000, LI-COR Biosciences, Lincoln, NE, USA); BlueEasy Pre-stained Protein Marker (MWP06, Nippon GENETICS EUROPE, Düren, Germany); and Color Pre-stained Protein Standard, Broad Range (P7712S, New England Biolabs, Ipswich, MA, USA). The intensity of the bands from at least 3 independent experiments was quantified by using the Gel Analyzer Plugin in IMAGE J software v1.53i (NIH, Bethesda, MD, USA). 

Animal tissue preparation and immunohistochemistry. On day 10, after injury, animals from both groups (the SCA group and the age-matched control group) were sacrificed. Brains were isolated and fixed in 4% PFA at 4 °C overnight, cryoprotected by incubation in sucrose gradient solutions (10%, 20%, and 30% in 0.2 M phosphate buffer pH 7.4), and then frozen in isopentane precooled to −80 °C. The brains were cut into 25 μm thick coronal slices, mounted on microscope glass slides, air-dried at RT, and stored at −20 °C until processed for immunohistochemistry. This cryopreserved histological material (brain coronal sections from control animals and animals with SCA) from previous work by coauthor S.D. [39] was used for ex vivo screening of SOX2 and SOX9 expression presented in this paper. Prior to immunostaining, the slides were kept at RT for 30 min. Heated citrate buffer (0.1 M, pH 6) was used for antigen retrieval. After washing twice for 5 min in 0.01 M PBS, the slides were incubated in a blocking solution containing 5% normal donkey serum (Sigma-Aldrich) in 0.1% Triton X-100 in PBS (PBT) for 1 h at RT. Sections were incubated with primary antibodies in a humid chamber at 4 °C overnight. The following primary antibodies were used for this experiment: mouse monoclonal anti-GFAP antibody (1:200; 73–240, UC Davis/NIH NeuroMab Facility); mouse polyclonal anti-vimentin antibody (1:300; M0725, Dako, Santa Clara, CA, USA); rabbit polyclonal anti-SOX2 antibody (1:100; 39823, Active Motif); rabbit monoclonal anti-Ki67 antibody (1:100; VP-K451, Vector Labs, Newark, CA, USA); and rabbit polyclonal anti-SOX9 antibody (1:100; ab5535, Merck). The next day, sections were washed three times in PBS and incubated for 2 h at RT with appropriate fluorescent dye-labeled secondary antibodies: donkey polyclonal anti-mouse lgG Alexa Fluor 488 (A-21202, Invitrogen, New York, NY, USA) and donkey polyclonal anti-rabbit lgG Alexa Fluor 555 (A-21428, Invitrogen), both diluted 1:250. The cell nuclei were stained with 4′,6-diamidino-2-phenylindole (DAPI) (Invitrogen) for 20 min at RT. After a final wash in PBS, the slides were mounted with a Mowiol mounting medium (Sigma-Aldrich). To test the specificity of the staining, brain sections incubated without primary antibodies were used. The staining was visualized using a Carl Zeiss AxioVision microscope (Zeiss, Jena, Germany). Images were captured using 10× and 40× objectives.

Immunocytochemistry (ICC). One-week-old and 4-week-old NT2/As were seeded on geltrex^®^-coated 18 mm coverslips placed in 12-well plates. The plating density of 1-week-old and 4-week-old NT2/A was 2 × 10^4^ cells per coverslip. The cells were fixed in 4% PFA in PBS for 20 min at RT, permeabilized with 0.2% Triton X-100 for 20 min, and blocked in PBT with the addition of 1% BSA and 5% normal goat serum (NGS) for 1 h at RT. Primary antibodies were diluted in PBT with 1% BSA and incubated overnight at 4 °C. Primary antibodies used for ICC were rabbit polyclonal anti-SOX2 (1:100; 39823, Active Motif); rabbit polyclonal anti-SOX9 (1:100; ab5535, Merck); rabbit polyclonal anti-Ki67 (1:300; ab15580, Abcam); mouse monoclonal anti-vimentin (1:300; sc-6260, Santa Cruz Biotechnology, Heidelberg, Germany); and rabbit polyclonal anti-S100β (1:100, S2644, Sigma-Aldrich). For immunostaining with rabbit polyclonal anti-GFAP (1:200; Z0334, Dako) and mouse monoclonal anti-SOX2 (1:300; MAB2018, R&D Systems, Minneapolis, MI, USA), after fixation with 4% PFA and washing with PBS, cells were subjected to antigen retrieval in citrate buffer pH 6.0 for 4 min at 75 °C. After cooling down, cells were washed with PBS, blocked with 5% BSA and 5% NGS in PBT for 1 h at RT, and incubated with primary antibodies diluted in PBT overnight at 4 °C.

The anti-SOX2 and anti-SOX9 antibodies were first labeled with biotinylated goat anti-rabbit IgG (1:500; BA-1000, Vector Laboratories, Newark, CA, USA) for 1 h at RT in PBS, followed by labeling with streptavidin conjugated with DyLight^®^ 488 (SA-5488, Vector Laboratories) diluted 1:500 in PBS containing 1% BSA for 1 h at RT. The anti-Ki67 antibody was labeled for 1 h at RT with an appropriate fluorescent dye-labeled secondary antibody: goat polyclonal anti-rabbit IgG (H+L) Alexa Fluor 488 (A-11034, Invitrogen) diluted 1:500 in PBT supplemented with 1% BSA. The anti-vimentin antibody was labeled with an appropriate fluorescent dye-labeled secondary antibody: goat polyclonal anti-mouse IgG (H+L) Alexa Fluor 546 (A-11030, Invitrogen). Actin filaments (F-actin) were stained by incubating cells for 1 h at RT in Phalloidin-iFluor 594 Reagent (ab176757, Abcam), diluted at 1:3000 in PBT supplemented with 1% BSA. Cell nuclei were stained with DAPI (Sigma-Aldrich). Immunofluorescence micrographs were taken with an Olympus BX51 fluorescence microscope using the Cytovision 3.1 software (Applied Imaging Corporation, San Jose, CA, USA) using 10×, 20×, 40×, and 60× objectives. 

Glutamate uptake assay. For the assessment of glutamate uptake by astrocytes, we used a glutamate assay kit (ab83389, Abcam). The total number of cells per sample was 3 × 10^6^, pooled from confluent 10 cm cell culture dishes. Cells were incubated with 2 mM glutamate (49449-100G, Sigma-Aldrich) in the Ca^2+^-containing Hank’s Balanced Salt Solution (HBSS) for 1 h. Afterward, cells were scraped from cell culture dishes and lysed in the assay buffer provided in the glutamate assay kit. Glutamate uptake was measured according to the manufacturer’s instructions using an Infinite 200 PRO microplate reader (Tecan). Uptake was normalized to the total amount of proteins [47] in the corresponding sample, measured by the Bradford assay (39222.02, Serva Electrophoresis GmbH). 

Statistical analyses. Statistical analyses were performed using the SPSS statistical software v20. The data represent the mean ± SEM of at least three independent experiments; *p*-values were calculated using the Student’s *t*-test. * *p* ≤ 0.05, ** *p* ≤ 0.01, and *** *p* ≤ 0.001.

## 3. Results

### 3.1. Expression of SOX2 and SOX9 in the Adult Cerebral Cortex

**Figure 1:**
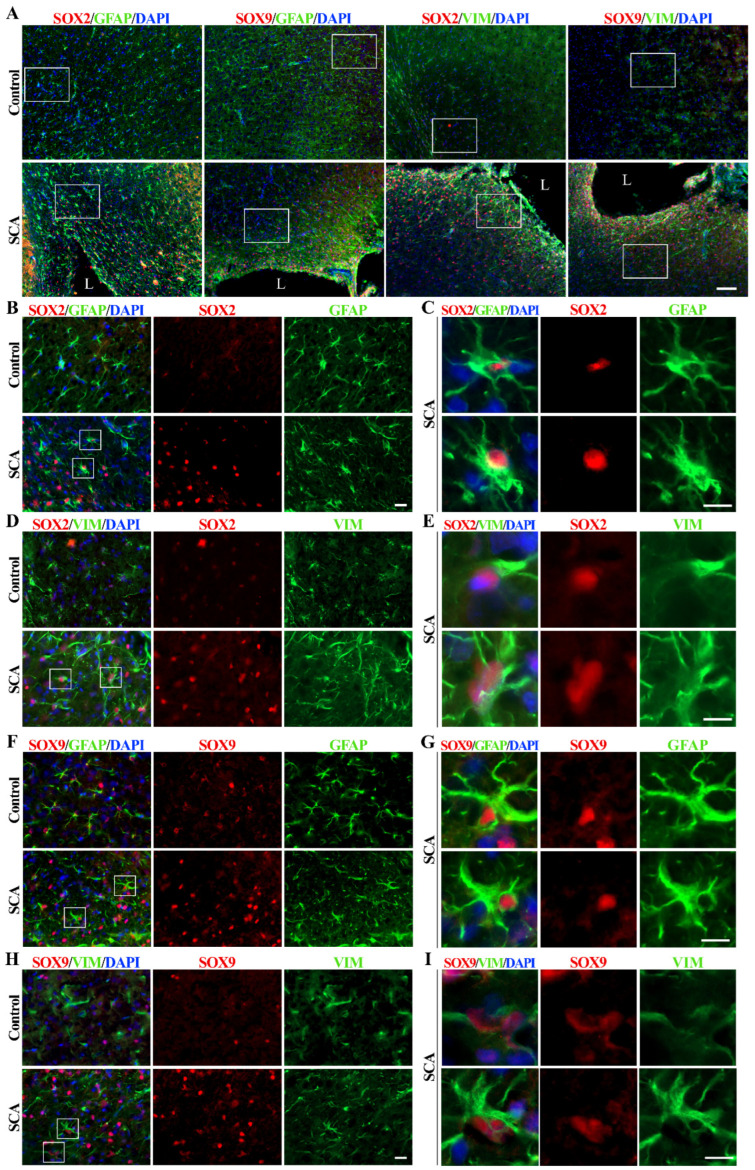
SOX2 and SOX9 expression alterations in cerebral cortex 10 days after SCA injury. (**A**) Representative micrographs of the right cerebral cortex immunofluorescently labeled for SOX2 and SOX9 (red), astrocytic markers GFAP and vimentin (VIM) (green), and nuclear marker DAPI (blue). In the control group, we detected rare and weak SOX2 and SOX9 immunoreactivity and GFAP-positive and rare vimentin-positive protoplasmic astrocytes in the cortex. The cortical injury resulted in the re-activation of astrocytes (detected by increased GFAP and vimentin immunoreactivity) as well as increased SOX2 and SOX9 signals, especially around the lesion site. Outlined regions, imaged under higher magnification, were presented in the following panels. The lesion site is annotated as L. (**B**,**D**) Higher magnification shows SOX2 (red) expression in the protoplasmic astrocytes of the control group and the reactive astrocytes of SCA rats. The outlined regions are presented under panels (**C**,**E**). (**C**,**E**) The framed areas show strong staining of SOX2+/GFAP+ or SOX2+/vimentin+ reactive astrocytes after SCA. Those cells predominantly have a nuclear expression of SOX2. (**F**,**H**) Higher magnification shows SOX9 immunoreactivity in astrocytes from the control and SCA cortex. The outlined regions are presented under panels (**G**,**I**). (**G**,**I**) Single cells from the outlined regions show strong SOX9 nuclear expression in reactive astrocytes labeled with GFAP or vimentin. The presented micrographs in Panel A were taken with the 10× objective. Micrographs in panels (**B**,**D**,**F**,**H**) were obtained using a 40× objective. Enlarged cells presented in panels (**C**,**E**,**G**,**I**) were imaged using 40× objective and cropped. Scale bars: (**A**)—100 µm; (**B**,**D**,**F**,**H**)—20 µm; (**C**,**E**,**G**,**I**)—10 µm.

To determine the relationship between SOX2 and SOX9 expression and astrocyte reactivation after injury, we examined the cerebral cortex of rats after SCA by immunohistochemical labeling (Figure 1 and Figure S1A,B). The injury site was made by suction ablation in the right hemisphere of SCA rats (Figure S1C). Intermediate filament markers GFAP and vimentin were used to identify reactive astrocytes. 

Sporadic protoplasmic astrocytes labeled with GFAP and expressing weak vimentin immunoreactivity were found in the cerebral cortex of control rats (Figure 1A). At the same time, the expression of SOX2 and SOX9 was low. A lesion of the right cerebral cortex resulted in the appearance of reactive astrocytes around the lesion site and increased expression of SOX2 and SOX9 proteins in SCA animals at day 10 post-injury (Figure 1A). It is noticeable that SOX2 immunoreactivity in the control group is restricted to the nuclei of a few cells, which are mainly GFAP-positive (Figure 1B) and weakly vimentin-positive (Figure 1D), indicating protoplasmic astrocytes with small bodies and thin processes.

Reactive astrocytes showed increased expression of intermediate filament proteins GFAP and vimentin, especially near the injury site (Figure 1), and increased expression of SOX2 (Figure 1A,B,D and Figure S1A) and SOX9 (Figure 1A,F,H and Figure S1B). Reactive astrocytes (Figure 1C,E,G,I) are characterized by an enlarged cell body and cell processes, as well as high expression of SOX2 and SOX9 proteins in the nucleus. SOX9 was detectable in sporadic cortical cells from control animals, particularly in GFAP-positive protoplasmic astrocytes (Figure 1F) and differentiated glial cells, with minimal vimentin expression (Figure 1H). Vimentin expression can be reactivated by brain injury [48], which is accompanied by astrocyte activation and dramatically increased SOX9 expression in the nucleus of reactive astrocytes in SCA animals 10 days after brain injury (Figure 1I).

Our results confirmed previously published data showing the up-regulation of SOX2 and SOX9 in reactive astrocytes after injury [33,36,37,49,50,51]. Also in accordance with previous reports [52,53], we detected increased proliferation in the tissue surrounding the injury site, as revealed by intense Ki67 immunoreactivity (Figure S1D). To investigate the role of SOX2 and SOX9 in this cellular process and other reactivation-related functional properties of astrocytes, we performed further studies in vitro using NT2/D1-derived astrocytes as a model system. 

### 3.2. Characterization of NT2/A at Different Stages of Maturity by Analysis of SOX2 and SOX9 mRNA and Protein Expression

**Figure 2:**
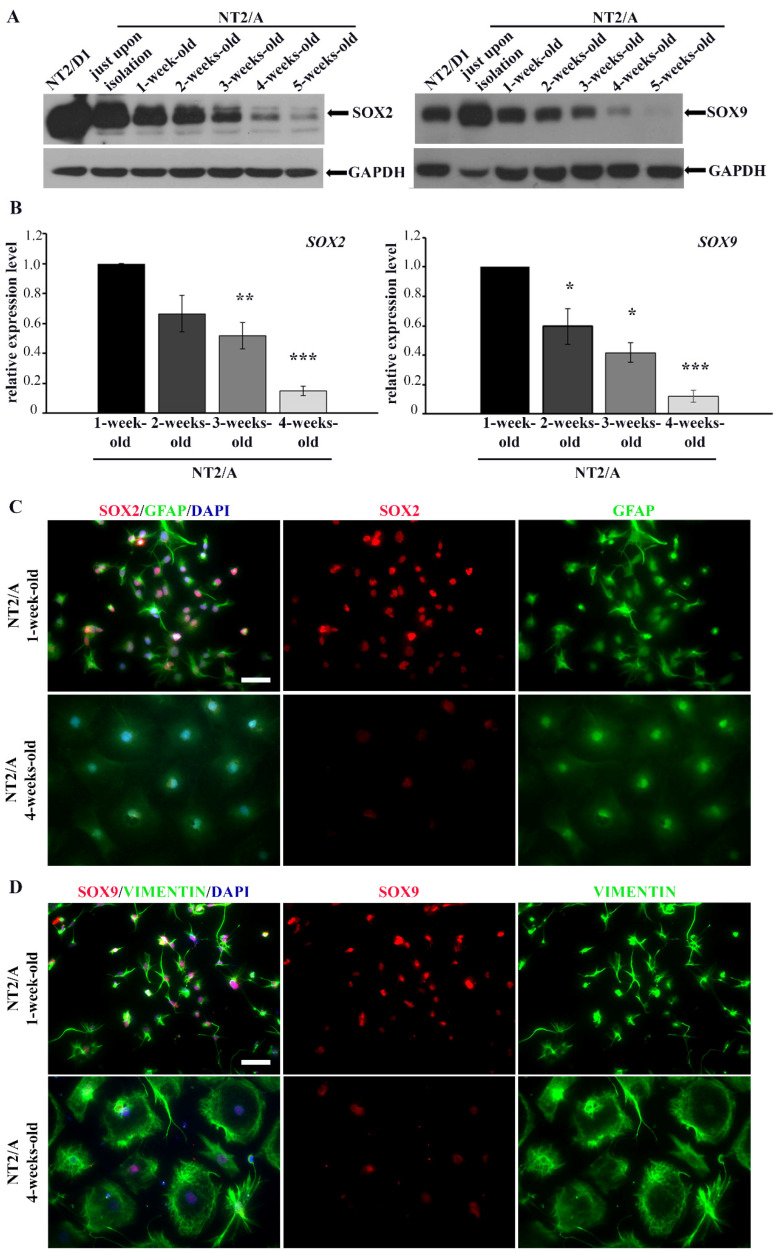
The analysis of SOX2/*SOX2* and SOX9/*SOX9* expression in different NT2/A maturation phases. (**A**) Western blot analysis of SOX2 and SOX9 protein expression in NT2/D1 cells and NT2/A at different maturation phases, from the moment when NT2/A were separated from NT2/D1-derived neurons (NT2/A just upon isolation) until the fifth week of their maturation in vitro. GAPDH was used as a loading control. The analysis was performed in three independent experiments, and the representative Western blot images are presented. (**B**) qPCR results showing relative *SOX2* and *SOX9* mRNA expression levels (expressed as fold change) in 2-, 3-, and 4-week-old NT2/A were calculated compared to the expression level in the 1-week-old NT2/A. Data were normalized to *GAPDH* as an endogenous control. Data represent means ± SEM (*n* = 3); *p*-values were calculated using Student’s *t*-test: * *p* ≤ 0.05, ** *p* ≤ 0.01; *** *p* ≤ 0.001. (**C**) Representative micrographs of 1-week-old and 4-week-old NT2/A immunofluorescently labeled with an antibody against GFAP (green) and an antibody against SOX2 (red). Cell nuclei were counterstained with DAPI (blue). (**D**) Representative micrographs of 1-week-old and 4-week-old NT2/A immunofluorescently labeled with an antibody against vimentin (green) and an antibody against SOX9 (red). Cell nuclei were counterstained with DAPI (blue). A clear distinction in morphology (visualized by GFAP and vimentin) and intensity of SOX2 and SOX9 nuclear expression between 1-week-old and 4-week-old NT2/A (merged and single channels) are notable in panels C and D. All micrographs have been taken using a 20× objective. Scale bar—50 μm.

During the process of maturation, NT2/A exhibit similar properties as primary astrocytes [27]. In our previous work [26] and in this work, we documented the maturation of NT2/A based on changes in cell morphology (Figure S2C,D), expression of astrocyte-specific markers (Figure 2C,D and Figure S2A,B) and a decrease in proliferation, confirming previously published data [27]. In this study, we analyzed the levels of SOX2 and SOX9 protein expression during NT2/A maturation in vitro (Figure 2A). Maturation of NT2/A was examined from the time when NT2/A were separated from NT2/N (NT2/A just upon isolation; see Materials and Methods) until the fifth week of their maturation, when they reached a nearly quiescent state [26,27]. We found that expression levels of both SOX2 and SOX9 gradually decreased as NT2/A maturation progressed. Indeed, the highest level of SOX2 and SOX9 expression was observed in NT2/A just upon their isolation (Figure 2A). However, when compared to progenitor NT2/D1 cells, the level of SOX2 expression was lower in just isolated NT2/A, while SOX9 expression was higher in astrocytes at this, the earliest stage of the maturation process. The gradual decline in *SOX2* and *SOX9* expression over the four weeks of the NT2/A maturation process was also confirmed at the mRNA level (Figure 2B).

To verify if the changes in expression levels of SOX2 and SOX9 correlate with the morphological changes of NT2/A that occur during the maturation process, we co-labeled immature (1-week-old) and mature (4-week-old) NT2/A with intermediate filament markers specific for astrocytes (GFAP and vimentin) and antibodies against SOX2 and SOX9 (Figure 2C,D). Additionally, for the same purpose, we used Phalloidin, which specifically binds to filamentous actin (F-actin), in co-staining with antibodies against SOX2 and SOX9 (Figure S2C,D). We clearly observed differences in cell morphology between 1-week-old and 4-week-old astrocytes (Figure 2C,D and Figure S2C,D). Our results showed that 1-week-old NT2/A cultures, consisting mostly of small fibrous or stellate astrocytes [26], exhibited high nuclear immunoreactivity for both SOX2 and SOX9. On the contrary, 4-week-old protoplasmic astrocytes, flat cells with short cytoplasmic extensions expanding from almost round cell bodies and characteristic of mature NT2/A cultures [26], showed low detectable nuclear positivity for SOX2 and SOX9 (Figure 2C,D and Figure S2C,D).

Our results demonstrated that, similar to primary astrocytes [35,54], high expression of SOX2 and SOX9 is typical for immature NT2/A that still has not reached a quiescent state. 

### 3.3. The Analysis of SOX2 and SOX9 Expression after In Vitro Induced Injury in Mature NT2/A

Sandhu et al. [27] demonstrated that both scratch-induced injury and treatment with the adenylate cyclase activator, forskolin, caused the reactivation of quiescent NT2/A, resulting in increased GFAP staining in cells with dramatically altered morphology. To investigate if the reactivation of mature NT2/A was accompanied by increased SOX2 and SOX9 expression, we used scratch as a mechanical stimulus to induce the reactive response of 4-week-old NT2/A.

**Figure 3:**
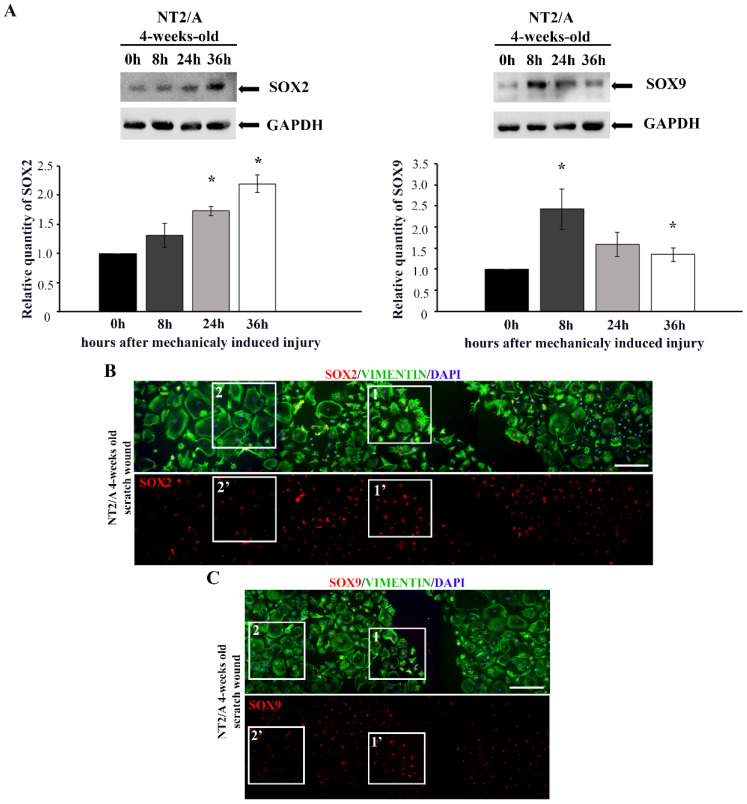
The analyses of SOX2 and SOX9 expression after in vitro injury-induced astrocyte reactivation. (**A**) Western blot analysis of SOX2 and SOX9 expression in 4-week-old NT2/A after scratch-induced injury. The levels of SOX2 and SOX9 expression were analyzed at the moment when the scratch injury was induced (time point 0 h) and 8, 24, and 36 h post-injury. GAPDH was used as a loading control. Quantified Western blot bands (average values of three independent experiments ± SEM) were presented as fold change compared to the corresponding values at time 0 h, and *p*-values were calculated using Student’s *t*-test: * *p* ≤ 0.05. (**B**,**C**) The confluent monolayer of 4-week-old NT2/A was scratched, fixed, and immunofluorescently labeled with an antibody against vimentin (green) and antibodies against SOX2 and SOX9 (red). Cell nuclei were counterstained with DAPI (blue). Five single images for SOX2 (**B**) and four single images for SOX9 (**C**) were taken in a sequence under the same imaging conditions and merged into a unified image showing a wide region around the injury. The squares marked as 1 and 1′ show the cells localized near the wounded region, characterized by high SOX2 (**B**) and SOX9 (**C**) expression and fibrous morphology. The squares marked as 2 and 2′ show the cells localized further away from the injury, characterized by low SOX2 (**B**) and SOX9 (**C**) expression and a large protoplasmic structure. Micrographs were taken using a 10× objective. Scale bar—200 μm.

The confluent monolayer of 4-week-old NT2/A was scratched, and SOX2 and SOX9 expression levels were analyzed by Western blot at 8, 24, and 36 h post-scratch-induced injury (Figure 3A). At all time points analyzed, an increase in the expression levels of both transcription factors was observed compared to time 0 h (the time point at which scratch injury was induced). Interestingly, the peak of SOX2 expression level was detected 36 h post-injury, with an approximately two-fold increase (2.2 ± 0.15) compared to the time point 0 h (Figure 3A). In the case of SOX9, the highest expression level was detected 8 h after scratch-induced injury, reaching approximately a 2.5-fold increase (2.5 ± 0.75) compared to time point 0 h (Figure 3A). 

Immunofluorescence labeling showed that cells localized closer to the wounded region (Figure 3B,C, boxed area 1, 1′) had higher SOX2 and SOX9 expression compared to the cells localized further away from the injury (Figure 3B,C, boxed area 2, 2′). Moreover, the cells in the vicinity of the scratched area had fibrous morphology characteristic of reactive astrocytes (visualized with vimentin in Figure 3B,C, boxed area 1), while cells away from the wounded area maintained protoplasmic shape, typical for the quiescent state (Figure 3B,C, boxed area 2). Thus, the significant SOX2 and SOX9 up-regulation was restricted to the mature NT2/A near the scratch region, and it was accompanied by the acquisition of morphology typical for reactive phenotypes, similar to the effect detected in vivo (Figure 1).

### 3.4. The Analysis of SOX2 and SOX9 Expression after Forskolin Treatment of Mature NT2/A

**Figure 4:**
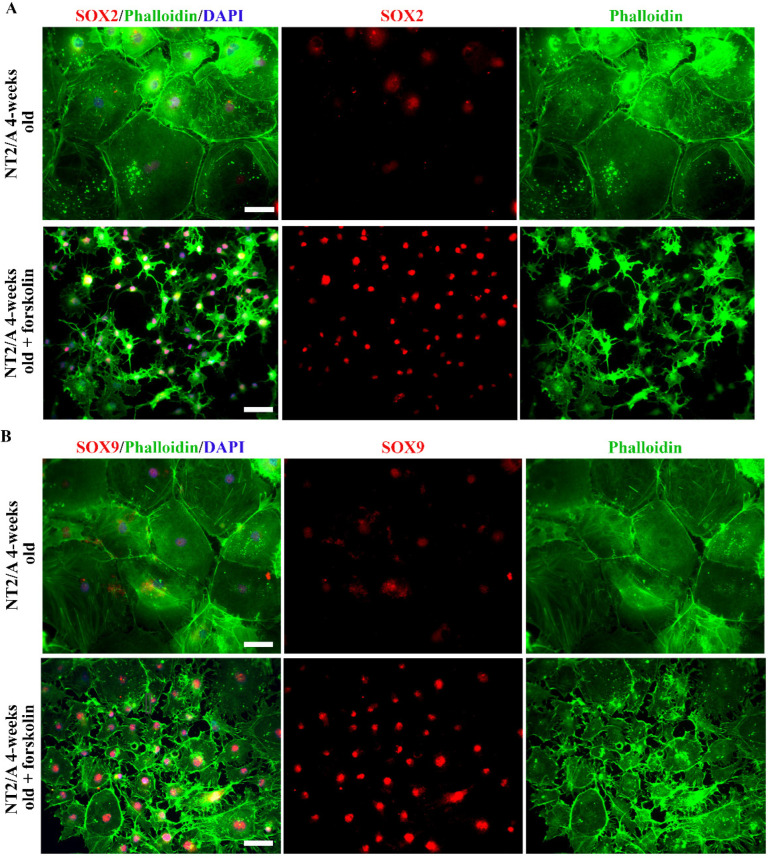
The analyses of SOX2 and SOX9 expression after forskolin-induced astrocyte reactivation. (**A,B**) Representative micrographs of the immunofluorescently labeled SOX2 (**A**) and SOX9 (**B**) (red), Phalloidin (green), and nuclear staining (DAPI, blue) in 4-week-old NT2/A after forskolin treatment. Forskolin treatment resulted in a well-defined morphological transformation of large 4-week-old protoplasmic NT2/A into stellate astrocytes (visualized by Phalloidin, merged and single channels) and intensification of SOX2 and SOX9 nuclear expression in the morphologically changed NT2/A (visualized in merged and single channels). Micrographs were taken using a 20× objective. Scale bar—50 μm.

The expression of SOX2 and SOX9 transcription factors was also examined in 4-week-old NT2/A after forskolin treatment (Figure 4). Immunofluorescence labeling carried out 24 h after the treatment demonstrated that forskolin-induced a well-defined morphological conversion of large protoplasmic mature NT2/A into stellate astrocytes (detected with Phalloidin, Figure 4A,B). In parallel to cytoplasmic reorganization, an intensification of SOX2 and SOX9 signals could be observed in the nuclei of morphologically altered NT2/A (Figure 4A,B). 

Thus, our results showed that both mechanical and chemical stimuli we applied to mimic some aspects of CNS trauma and induce astrocyte reactivation in vitro caused an increase in SOX2 and SOX9 expression, a similar effect that we observed in astrocytes in an animal model of SCA (Figure 1).

### 3.5. The Analyzes of Proliferation and Migration Capabilities after SOX2 and SOX9 Overexpression in Mature NT2/A

Reactive astrocytes undergo profound functional changes, including increased proliferation and migration towards the site of injury [15,19]. These injury-induced phenotypic changes of mature astrocytes are also recognized in astrocyte precursors during CNS development [18,19,55], but the underlining molecular mechanisms are not well understood.

**Figure 5:**
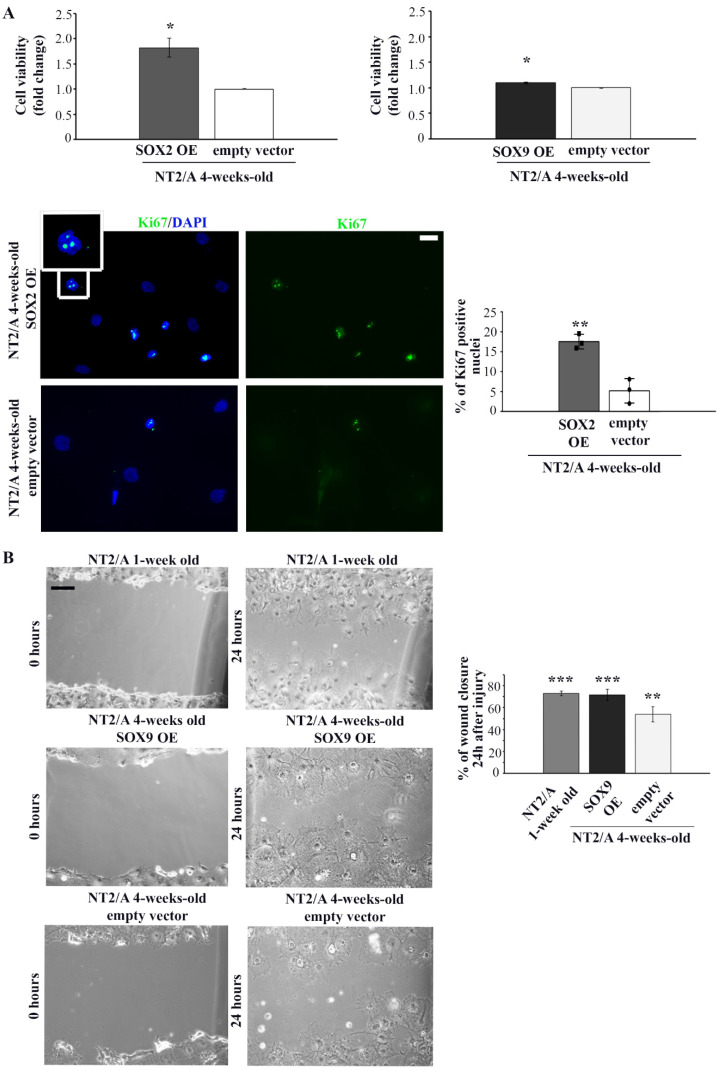
The analysis of proliferation and migration capabilities of 4-week-old NT2/A transduced with *SOX2* and *SOX9* expression vectors. (**A**) Relative cell viability of 4-week-old NT2/A after transduction with *SOX2* or *SOX9* expression vectors (indicated as SOX2 OE and SOX9 OE, where OE stands for overexpression) was measured by MTS assay and compared with the viability of cells transduced with the corresponding empty vector (control). Data presented are means ± SEM (*n* = 3); *p*-values were calculated using Student’s *t*-test: * *p* ≤ 0.05. Representative images of Ki67 immunostaining (green) of 4-week-old NT2/A transduced with *SOX2* expression vector or empty vector (control) counterstained with nuclear marker DAPI (blue). The boxed region is magnified to show the nuclear localization of Ki67. Micrographs were taken using a 60× objective. Scale bar—20 µm. The percentage of Ki67+ cells presented in the chart was calculated relative to the total number of DAPI-labeled nuclei in three independent experiments. Data represent means ± SEM, and *p*-values were calculated using Student’s *t*-test: ** *p* ≤ 0.01. (**B**) Representative phase contrast images of wound closure experiments. The migratory capability of 1-week-old NT2/A and 4-week-old NT2/A transduced with *SOX9* expression vector or empty vector (control) was measured 24 h after scratch injury. Scale bar—100 µm. The statistical analysis of the percentage of wound closure was obtained from three independent experiments. Data represent means ± SEM, and *p*-values were calculated using Student’s *t*-test: ** *p* ≤ 0.01, *** *p* ≤ 0.001.

To investigate if SOX2 and SOX9 contribute to some of the reactivation-related cellular processes, we overexpressed each of these transcription factors in 4-week-old NT2/A and analyzed the effects on cell viability, proliferation, and migration capabilities (Figure 5). We transduced 4-week-old NT2/A with the constructs expressing *SOX2* and *SOX9* and analyzed their expression five days after transduction at the mRNA (Figure S3A) and protein (Figure S3B) level. 

After confirmation of successful transduction, we examined whether up-regulation of SOX2 and SOX9 affected the viability of 4-week-old NT2/A. The viability of mature NT2/A was analyzed five days after transduction by the MTS assay (Figure 5A). While up-regulation of SOX2 led to an increase in cell viability (1.82 ± 0.19) compared to NT2/A control, overexpression of SOX9 had almost no effect (1.09 ± 0.02). To determine if increased cell viability of the NT2/A after transduction with the *SOX2* expression vector was, at least in part, caused by the increase in the cell proliferation rate of these almost quiescent cells [26,27], we analyzed the expression of Ki67, a nuclear protein associated with cellular proliferation (Figure 5A, lower panel). Our results indicated that the number of Ki67-positive NT2/A cells (Ki67+) increased due to up-regulation of SOX2. In particular, NT2/A overexpressing SOX2 had 17% of Ki67+ cells, compared to only 5% of mitotically active NT2/A control cells (Figure 5A).

Next, we investigated whether SOX2 and SOX9 overexpression influenced the migratory capability of 4-week-old NT2/A by performing the classical scratch wound healing assay (Figure 5B and Figure S4). No substantial difference in the migratory capability of 4-week-old NT2/A was detected when these cells were transduced with the SOX2 expression vector (Figure S4). On the other hand, 72% of the wounded area was closed due to the migration of NT2/A overexpressing SOX9, compared to 54% of wound closure in the NT2/A control cells (Figure 5B). Similarly, 1-week-old NT2/A, characterized by high SOX9 expression (Figure 2A), covered 73% of the wounded surface (Figure 5B). Our results suggested that up-regulation of SOX9 in 4-week-old NT2/A cells resulted in an increase in migration rate of almost 20% compared to control. 

In summary, our results showed that SOX2 overexpression increased the proliferation rate of mature NT2/A, while SOX9 overexpression had a positive effect on their migratory capacity. 

### 3.6. The Analysis of SOX9 Target Gene Expression and the Capacity for Glutamate Uptake of Mature NT2/A Overexpressing SOX9

To determine if the up-regulation of SOX9 in mature NT2/A reactivates the same transcriptional regulatory cascade that acts in the initiation of gliogenesis [34], we analyzed the expression of SOX9 target genes, which are known to regulate key aspects of astro-glial precursor physiology during development [34]. We particularly focused our attention on NFIA because this transcription factor plays a key role in the onset of gliogenesis and, in a complex with SOX9, co-regulates a number of genes that are induced after glial initiation, including *Apcdd1*, which has a crucial migratory function during astro-gliogenesis [34].

**Figure 6:**
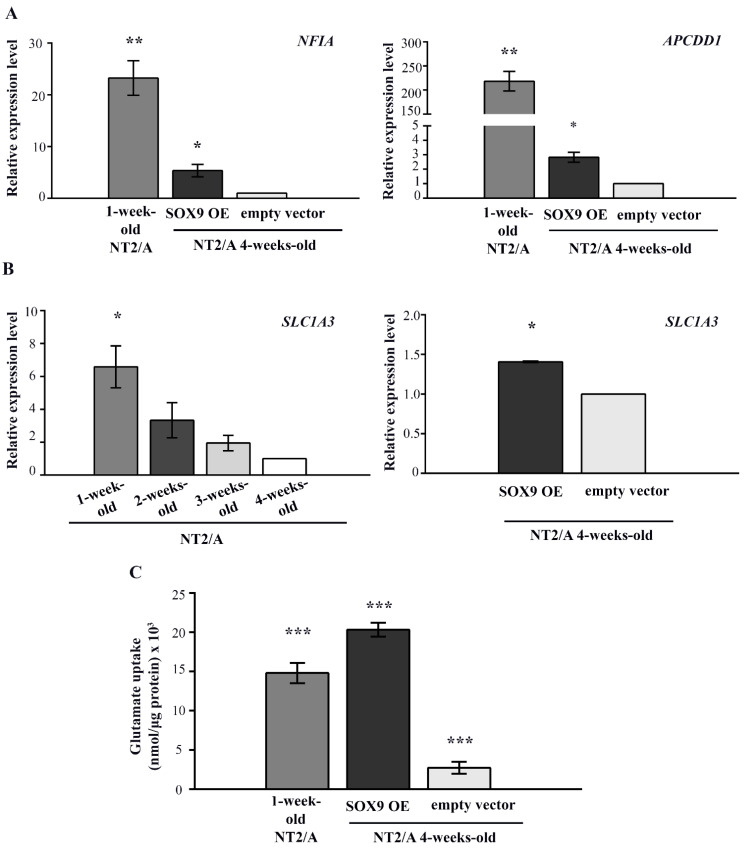
The analysis of SOX9 target gene expression and the capacity for glutamate uptake in 4-week-old NT2/A transduced with *SOX9* expression vector. (**A**) qPCR results showing relative *NFIA* and *APCDD1* gene expression (presented as fold change) in 1-week-old NT2/A and 4-week-old NT2/A transduced with the SOX9 expression vector were calculated compared to the expression level of these transcription factors in the 4-week-old NT2/A transduced with the empty vector (control). Data were normalized to *GAPDH* as an endogenous control. Data represent means ± SEM (*n* = 3), and *p*-values were calculated using Student’s *t*-test: * *p* ≤ 0.05 and ** *p* ≤ 0.01. (**B**) The relative level of glutamate transporter *SLC1A3* expression was analyzed by qPCR at the mRNA level during the maturation process of NT2/A and after the transduction of 4-week-old NT2/A with the SOX9 expression vector. The relative level of *SLC1A3* expression (expressed as a fold change) in 1-week-old, 2-week-old, and 3-week-old NT2/A was calculated compared to the corresponding value of 4-week-old NT2/A. The relative level of *SLC1A3* expression (expressed as fold change) in 4-week-old NT2/A transduced with the SOX9 expression vector was calculated compared to the expression level of this glutamate transporter in the 4-week-old NT2/A transduced with the empty vector (control). Data were normalized to *GAPDH* as an endogenous control. Data represent means ± SEM (*n* = 3), and *p*-values were calculated using Student’s *t*-test: * *p* ≤ 0.05. (**C**) The capacity for glutamate uptake in 4-week-old NT2/A transduced with the SOX9 expression vector and in the control group was analyzed by a glutamate uptake assay. Uptake was normalized to the total amount of proteins in the corresponding sample and expressed as nmol/µg protein [47]. Data represent the mean ± SEM of at least three independent experiments; *p*-values were calculated using Student’s *t*-test: *** *p* ≤ 0.001.

In our model system, the highest gene expression of *NFIA* and *APCDD1* was observed in immature (1-week-old) NT2/A (Figure 6A). Overexpression of SOX9 in mature NT2/A resulted in a significant five-fold increase in *NFIA* expression (5.355 ± 1.2) and reactivation of *APCDD1* gene expression to 2.5-fold (2.55 ± 0.34) compared to the 4-week-old NT2/A transduced with an empty vector (Figure 6A).

Since SOX9 is also known to induce the expression of glutamate transporter GLAST/EAAT1 during gliogenesis [34], which is known to be essential for the maintenance of synaptic glutamate homeostasis [5], we analyzed its expression during the maturation process of NT2/A and after the transduction of 4-week-old NT2/A with the *SOX9* expression vector (Figure 6B). Our result showed a gradual decrease in *SLC1A3* gene expression (coding for the GLAST/EAAT1 protein) during the course of NT2/A maturation. Interestingly, although transduction of 4-week-old NT2/A with the *SOX9* expression vector induced only a small increase (1.41 ± 0.01) in *SLC1A3* expression compared to the NT2/A control, we detected a considerably high capacity of these cells for glutamate uptake (Figure 6B,C). Interestingly, this capability for glutamate uptake is not directly related to *SLC1A3* expression levels. Although 1-week-old NT2/A had almost five-fold higher *SLC1A3* expression (6.58 ± 1.27) than 4-week-old NT2/A overexpressing SOX9 (1.41 ± 0.01), their ability for glutamate uptake was significantly lower compared to NT2/A overexpressing SOX9 (14.8 nmol/µg and 20.3 nmol/µg, respectively). 

Our results demonstrated that the up-regulation of SOX9 in mature NT2/A reactivates similar molecular mechanisms acting during the initiation of gliogenesis and increases the glutamate uptake capability of 4-week-old NT2/A.

## 4. Discussion

SOX transcription factors, as a part of complex regulatory networks, govern diverse cellular processes during development, including the maintenance of stem cell pluripotency, cell proliferation, cell fate decisions, germ layer formation, and terminal differentiation of cells into tissues and organs [56,57]. SOX2 and SOX9 transcription factors are expressed during early embryonic development, and it is known that they drive the expression of phenotype-specific genetic programs during neurodevelopment [28]. One of the well-known roles of SOX2 is the maintenance of stem cell pluripotency [57,58,59]. This transcription activator is also among the earliest expressed transcription factors in neural stem cells, with a key role in defining early neural lineages and brain development [60,61]. The SOX9 transcription factor has been reported to play a crucial role in triggering the switch from a neurogenic to a gliogenic program in the developing mouse spinal cord [34,35]. In the adult brain, the expression of both SOX2 and SOX9 transcription factors is mostly restricted to the neurogenic regions, where they have important roles in the regulation of adult neurogenesis [56,62,63]. However, it is becoming increasingly clear that *SOX* genes also play additional roles in adult tissue homeostasis and regeneration [33,64,65], but this field of research is less explored. Several studies have shown that SOX2 and SOX9 are re-expressed in reactive astrocytes after injury [33,36,37], but their roles in these cells are still not fully understood. Although the pathological contexts in which astrocyte reactivity occurs can markedly vary [66], targeting specific genes that modulate the functional properties of reactive astrocytes could have important effects on the outcome of neural injury [33,67]. Astrocyte-targeted therapeutic approaches are among the potential neuroprotective strategies that hold great promise for the treatment of different CNS pathologies [12,68,69,70]. Accordingly, deciphering the mechanisms governed by SOX2 and SOX9 in reactive astrocytes is critical for guiding astrocyte-targeted therapies.

SOX2 expression has been shown to decrease in adults compared to the embryonic stage in mouse neocortex. However, astrocytes retain SOX2 expression even after they acquire glial fate and until they become quiescent [36]. In the cerebral cortex of our control rat model, we detected low expression of SOX2 and SOX9, except for a few sporadic protoplasmic astrocytes that were GFAP positive with minimal vimentin expression (Figure 1A,F,H). Due to its very low expression or the rare and random presence of cells co-labeled with SOX9 and GFAP in control animals (Figure 1A,F), we cannot agree with the recognition of SOX9 as an astrocyte-specific nuclear marker as reported by others [37,71]. Nevertheless, we cannot exclude that this discrepancy could be based on species specificity, considering that our screening of SOX9 expression was conducted on rat cerebral cortex, whereas the data reported by others [37,71] refer to human and mouse adult brain sections.

In vitro, our results showed a gradual decrease in SOX2 and SOX9 during the differentiation process from NT2/D1 to NT2/A (Figure 2A). We detected the highest expression of SOX2 in undifferentiated parental NT2/D1 cells, which is expected since these cells resemble early embryonic stem cells [40]. Gradual down-regulation of SOX2 at protein and mRNA levels (Figure 2A,B) was observed by the fourth week of in vitro maturation when NT2/A reached quiescence [26,27]. Similarly, we detected the highest SOX9 expression in astrocytes at the earliest stage of the NT2/A maturation process (just upon isolation, Figure 2A) and a gradual decrease in its expression in maturing astrocytes at protein and mRNA levels (Figure 2A,B).

Several studies have shown the re-expression of transcription factors SOX2 and SOX9 in reactive astrogliosis occurring after injury, middle cerebral artery occlusion, multiple ministrokes, and neurodegeneration [33,36,37]. Our initial screening of these transcription factors in rats after an induced lesion of the cerebral cortex showed increased expression of SOX2 and SOX9 in reactive astrocytes, distinguished by increased GFAP expression and vimentin reactivation after SCA (Figure 1). A similar observation was made in a study by Bani-Yaghoub M. et al. [36], which demonstrated SOX2 re-expression in the quiescent astrocytic cultures that resumed proliferation after injury. Our observation was also confirmed in vitro. We demonstrated that the reactivation of mature NT2/A was associated with increased SOX2 expression when we applied mechanical and chemical stimuli to induce the reactive response of 4-week-old NT2/A (Figure 3). Moreover, our gain-of-function studies showed that SOX2 overexpression leads to an increase in NT2/A proliferative potential (Figure 4A), demonstrating the direct involvement of the SOX2 transcription factor in the regulation of reacquisition of proliferation of almost quiescent NT2/A. Since overexpression of SOX9 in almost quiescent 4-week-old NT2/A did not cause changes in cell viability (Figure 4A) or in the number of Ki67-positive nuclei, we can assume that SOX9 overexpression does not affect cell proliferation. Our results differ from those of Xia M. and Zhu Y. [72], who showed that Sox9, which was increased by adenosine triphosphate (ATP) in cultured spinal cord astrocytes, was involved in the increased proliferation and cell viability, while Sox2 was not correlated with astrocytic proliferation stimulated by ATP. We can assume that the results published by Xia M. and Zhu Y. [72] might be specifically related to the proliferation mechanism of ATP-stimulated spinal cord astrocytes. Indeed, depending on the timing and context, different stimuli released due to tissue damage could activate different intracellular signaling pathways in reactive astrocytes that ultimately lead to different outcomes, as reported for neuroinflammation [73,74]. 

Several studies indicated that gene expression profiles of reactive astrocytes correspond to those specific for astrocyte precursors and neonatal astrocytes during early brain development [10,75]. On this basis, in this study, we examined whether up-regulation of SOX9, detected by us and others in reactive astrocytes [37], reactivates the same transcriptional regulatory cascade that acts in the initiation of gliogenesis. Data from the embryonic spinal cord of chicks and mice showed that SOX9 directly regulates the expression of the transcription factor NFIA. Subsequently, SOX9 and NFIA form a complex that co-activates multiple genetic programs that regulate the activities of astroglial precursors, including the expression of the *Apcdd1* gene, which plays a crucial role in migration during astro-gliogenesis [34]. Our gain-of-function studies have shown that SOX9 overexpression in mature NT2/A results in increased expression of the *NFIA* and *APCDD1* genes. However, the expression of these genes was significantly lower in SOX9-transduced 4-week-old NT2/A compared to 1-week-old NT2/A, which we used as a positive control (Figure 5A). This result could be explained by the fact that, despite the efficient transduction of 4-week-old NT2/A with the *SOX9* expression vector, we did not achieve the endogenous level of SOX9 expression detected in 1-week-old NT2/A (Figure S2). The study by Peng Kang et al. [34] has indicated that APCDD1 plays a key role in the migration of astrocyte precursor populations, probably through an association with the Ras homologous (Rho) protein family GTPases. Accordingly, the increase in migratory potential of mature NT2/A that we detected after SOX9 overexpression (Figure 4B) could be attributed to the elevated expression of the *APCDD1* gene.

Both resumed proliferation ability and astrocyte migration are important regulators of glial scar formation. These required properties enable reactive astrocytes to interact and form a barrier that isolates inflammatory cells, containing an otherwise harmful inflammatory response to the site of injury [19,76]. Thus, deciphering the molecular mechanisms that underlie these highly orchestrated processes is of great importance.

Glutamate uptake in the CNS is primarily mediated by astrocytic high-affinity glutamate transporters GLT1/EAAT2 and GLAST/EAAT1 [5,6,7]. The reduced expression of GLT1/EAAT2 and GLAST/EEAT1 proteins (coded by Solute Carrier Family 1 Member 2 (*SLC1A2)* and *SLC1A3* genes, respectively) in astrocytes leads to glutamate-mediated excitotoxicity that causes neuronal damage, a condition that has been observed in the brain during aging, various pathologies such as traumatic brain injury, and numerous neurodegenerative diseases [6,7,77]. Indeed, among the various phenotypic changes exhibited by reactive astrocytes with detrimental effects on surrounding neural tissue, down-regulation of glutamate transporters has also been reported [21]. Similar to our results regarding *SLC1A3* expression, Sandhu et al. [27] showed low expression of both *SLC1A2* and *SLC1A3* in 4-week-old NT2/A. However, the expression pattern of both glutamate transporters was comparable to the pattern detected in primary cultures of human fetal astrocytes, and the authors also demonstrated their functional activity. They attributed the low expression of these glutamate transporters to the absence of neurons in pure cultures of NT2/A [27]. In agreement with previously published data [34], suggesting that SOX9-driven up-regulation of NFIA leads to the ectopic induction of GLAST/EEAT1, we detected an increase in the expression of this glutamate transporter in 4-week-old NT2/A after SOX9 overexpression (Figure 5B). However, *SLC1A3* expression was lower in 4-week-old NT2/A transduced with the *SOX9* expression vector than in 1-week-old NT2/A, which correlates with the difference in *NFIA* expression between these cells (Figure 5A,B). Accordingly, the gradual decrease in *SLC1A3* expression during the NT2/A maturation process (Figure 5B) might be related to the steady decrease in SOX9/*SOX9* expression in these cells (Figure 2A,B). Moreover, mature NT2/A transduced with the *SOX9* expression vector demonstrated higher glutamate uptake ability than control cells (cells transduced with an empty vector) and 1-week-old NT2/A (Figure 5). We did not detect an increase in *SLC1A2* expression in 4-week-old NT2/A after SOX9 overexpression, but we can assume that SOX9 overexpression in 4-week-old NT2/A triggered additional molecular mechanisms besides *SLC1A3* up-regulation that promoted augmented glutamate uptake.

In accordance with previously published data indicating that a battery of genes associated with glial scar formation are annotated SOX2 and SOX9 targets [33,78], our findings demonstrated that these transcription factors govern important reactivation-related functional properties of astrocytes. Although additional research is needed to uncover components of multiple signaling pathways regulated by these transcription factors in reactive astrogliosis, our findings suggest that the SOX2 and SOX9-dependent pathways may be specifically targeted in order to manipulate the fate of reactive astrocytes towards neural tissue regeneration and recovery. This is of great importance as reactive astrocytes are increasingly recognized as potential targets for novel therapeutic strategies in traumatic brain injury (TBI), contributing to post-traumatic tissue repair and synaptic remodeling [79]. As a major cellular component of the glial scar, reactive astrocytes may produce anti-regenerative molecules such as chondroitin sulfate proteoglycans (CSPGs) or pro-regenerative molecules such as laminin and fibronectin. Interestingly, SOX9 levels might be crucial for the balance between pro- and anti-regenerative extracellular matrix molecules produced by astrocytes [80]. Moreover, conditional SOX9 ablation reduced CSPGs after spinal cord injury and resulted in increased neuroplasticity and better locomotor recovery in mice [41,42]. Our results suggest that SOX9 is involved in astrocyte migration, most likely via activation of the same transcriptional regulatory cascade that acts in gliogenesis. This reacquired property of astrocytes is characteristic of the scar-forming stage after severe CNS damage [19]. Thus, deeper insight into the underlying mechanism of this cellular process may provide a therapeutic window for the control of astrogliosis. Glutamate is the major excitatory neurotransmitter in the CNS [2], and its impaired clearance from the synaptic space can cause glutamate excitotoxicity, neuronal hyperexcitation, and damage [81]. Our results indicate that SOX9 overexpression promotes glutamate uptake, which could have potential therapeutic importance since glutamate-mediated excitotoxicity has been observed in various CNS pathologies, including TBI [6,7,65]. Among the genes annotated to SOX2-bound elements, numerous have been associated with astrogliosis and reactive astrocyte proliferation after TBI or other brain diseases, such as Nr2e1, Mmd2, Wnt7a, and Akt2 [33]. This is consistent with our results showing that SOX2 overexpression leads to an increase in astrocyte viability and proliferation. Moreover, astrocyte-specific deletion of Sox2 in adult mice greatly reduced the glial response and promoted functional recovery after TBI in mice [33]. These results suggest that SOX2 regulates astrocyte function by directly targeting components of various signaling pathways and that this transcription factor or its target genes are potential targets for therapeutic intervention in TBI.

## 5. Conclusions

Although SOX transcription factors are known to regulate the expression of phenotype-specific genetic programs during neurodevelopment [28], their role in astrocytes beyond early development has not been extensively studied. Accordingly, we focused our attention on transcription factors SOX2 and SOX9, which have been shown to be re-expressed in reactive astrocytes [36,37]. We demonstrated that they govern important reactivation-related functional properties of astrocytes. In accordance with the previous findings indicating a link between astroglial SOX2 expression and cell cycle [36], we have demonstrated the direct involvement of SOX2 in the reacquisition of proliferation in almost quiescent NT2/A. To the best of our knowledge, we report for the first time the contribution of SOX9 in promoting migration in post-mitotic astrocytes, probably by reactivation of the same transcriptional regulatory network present during gliogenesis. Our studies also showed increased glutamate uptake upon SOX9 overexpression in mature NT2/A, most likely due to up-regulation of *SLC1A3* and some additional yet-to-be-identified molecular mechanisms. Although more comprehensive studies are needed to fully understand the exact roles of these transcription factors in the complexity of reactive astrogliosis, our results imply that modulation of SOX gene expression may change the functional properties of astrocytes. Our findings could contribute to the future development of novel potential therapeutic strategies for neural tissue regeneration and recovery.

## Data Availability

The data presented in this study are available in the article and Supplementary Material.

## Supplementary Materials

The following supporting information can be downloaded at: https://www.mdpi.com/article/10.3390/biomedicines12040796/s1, Figure S1: Images of the lesion of the right cerebral cortex after sensorimotor cortex ablation (SCA) and isolated whole brain of control and operated animals. Figure S2: Characterization of NT2/A by the expression of astrocyte specific markers and morphological changes. Figure S3: The analysis of transduction efficiency. Figure S4: The analysis of migratory capacity of 4-week-old NT2/A transduced with SOX2 expression vector by scratch wound healing assay.
