# Supplementary material for: The Role of SOX2 and SOX9 Transcription Factors in the Reactivation-Related Functional Properties of NT2/D1-Derived Astrocytes
Source: Biomedicines. 2024 Apr 3;12(4):796. doi: 10.3390/biomedicines12040796 (PMC11048103; doi:10.3390/biomedicines12040796)
Supplement: Supplementary file 1 [file biomedicines-12-00796-s001.zip › biomedicines-2821183-supplementary.pdf]

Figure S1- Images of the lesion of the right cerebral cortex after sensorimotor cortex ablation (SCA) and isolated whole brain of control and operated animals

(**A**) Representative composite image of the lesion site, consisting of five merged consecutive images after staining with SOX2 (red) and DAPI as nuclear marker (blue). In the right cerebral cortex (RCX), sensorimotor cortex lesion (SCA) caused extensive damage (L-lesion site). Scale bar: 200 µm. (**B**) Representative composite image of the lesion site consisting of three merged consecutive images after staining with SOX9 (red), GFAP (green) and DAPI as nuclear marker (blue). In the right cerebral cortex, SCA caused extensive lesion (L). Scale bar: 200 µm (**C**) Representative whole brain images of control rats and rats that underwent SCA. The lesion site is clearly visible in the right hemisphere of the SCA rats (arrow). (**D**) Representative composite image of a coronal brain section of SCA animals after staining with Ki67, a marker for proliferating cells. Sporadic and rare Ki67-reactive cells are observed in the right cortex (RCX) away from the injury site (L), but SCA induces increased proliferation in the tissue surrounding the injury site. Scale bar: 100 µm.

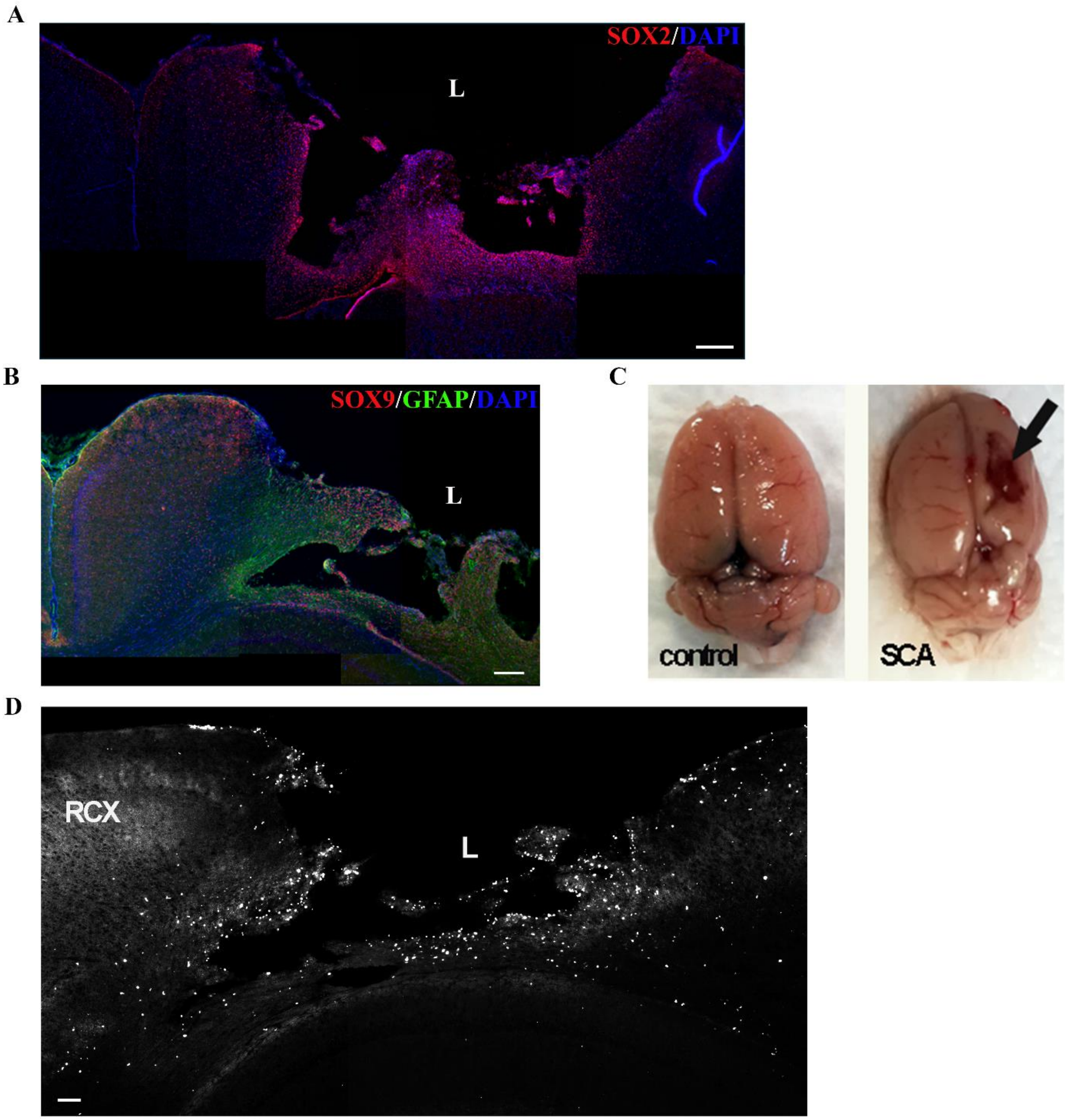

Figure S2- Characterization of NT2/A by the expression of astrocyte specific markers and morphological changes.

(**A**) Representative Western blot images show GFAP expression in NT2/A during the maturation process, from the 1st until the 4th week of its maturation *in vitro*. The expected band at 50 kDa (arrow) is the dominant band in the first three weeks of maturation. When NT2/A have reached 4th week of maturation, other bands of lower molecular weight come to the fore. GAPDH was used as a loading control (**B**) Representative micrographs of 1- and 4-weeks-old NT2/A immunoflourescently labelled with astrocytic markers vimentin (red) and S100β (green). Cell nuclei were counterstained with DAPI (blue). Scale bar: 50 µm. (**C** and **D**) Representative micrographs of 1-week-old and 4-weeks-old NT2/A immunofluorescently labelled with Phalloidin (green) and antibodies against SOX2 and SOX9 (red). Cell

nuclei were counterstained with DAPI (blue). Clear differences in morphology (visualized by Phalloidin, merged channels) and intensity of expression of SOX2 (C) and SOX9 (D) in the nuclei 1-week and 4-weeks-old NT2/A are visible in merged and single channels. Scale bar: 50 µm.

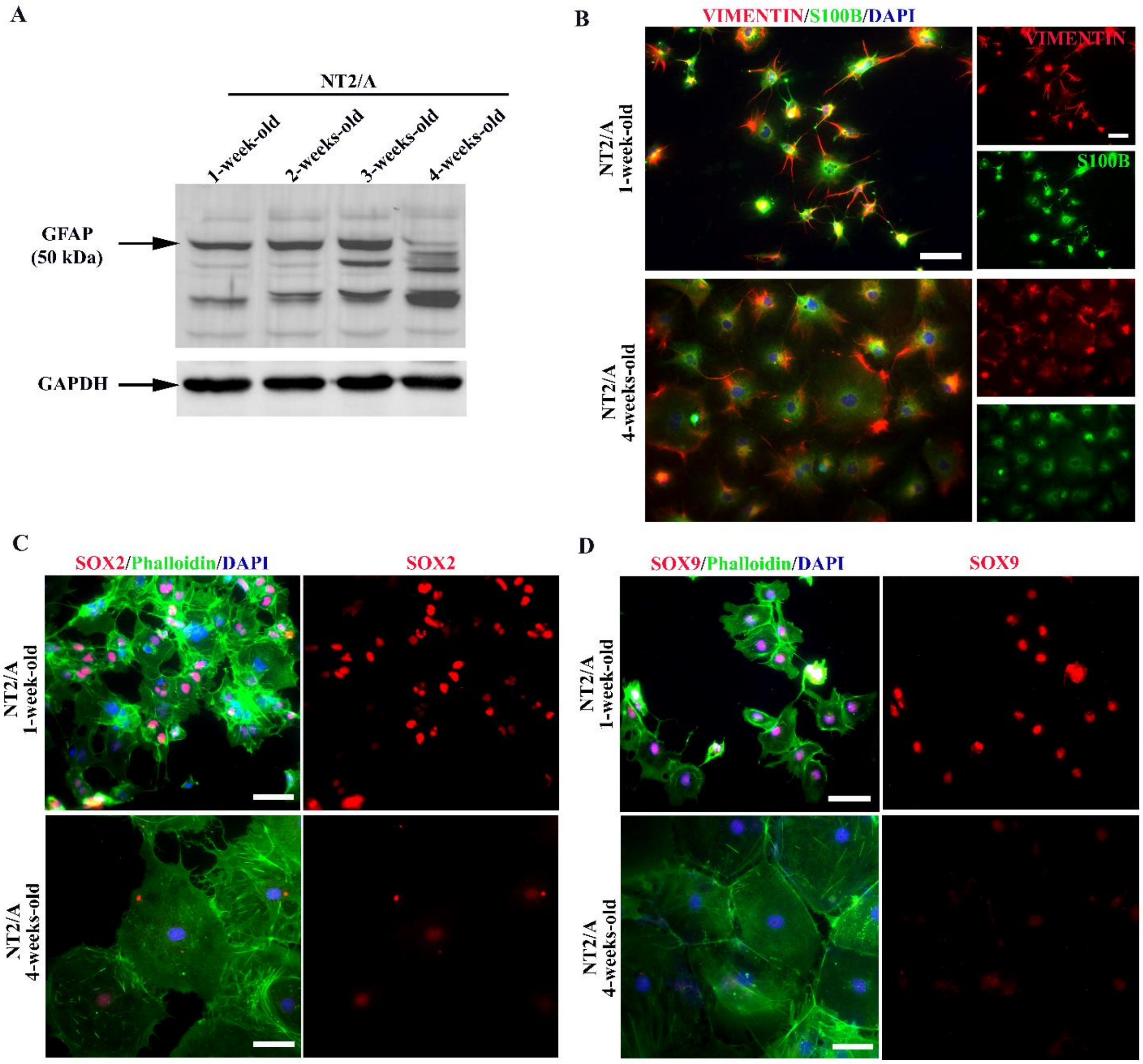

Figure S3-The analysis of transduction efficiency.

(A) qPCR results show the relative *SOX2* and *SOX9* mRNA expression levels (expressed as a fold increase) in 1-week-old NT2/A and 4-weeks-old NT2/A transduced with SOX2 or SOX9 expression vectors compared to the corresponding values in the 4-weeks-old NT2/A transduced with corresponding empty vectors (control). Data were normalized to *GAPDH* as endogenous control. Data represent means ± SEM (n = 3), *p*-values were calculated using Student's t-test, * $p \leq 0.05$, ** $p \leq 0.01$. The expression ofboth transcription factors was significantly increased upon transduction, in the case of SOX2 by 1.95±0.036

and in the case of SOX9 3.87±0.35, compared to the cells transduced with the empty vector (control NT2/A). However, the endogenous expression of these transcription factors characteristic of immature 1-week-old NT2/A was not reached. **(B)** Representative Western blot images show SOX2 and SOX9 overexpression (OE) in 4-weeks-old NT2/A after transduction with SOX2 and SOX9 expression vectors compared to 4-weeks-old NT2/A transduced with the corresponding empty vectors. TUBULIN or GAPDH were used as loading controls. Transductions were considered successful if the overexpression of SOX2 and SOX9 in 4-weeks-old NT2/A could be confirmed at both mRNA and protein levels; only these samples were used for further experiments.

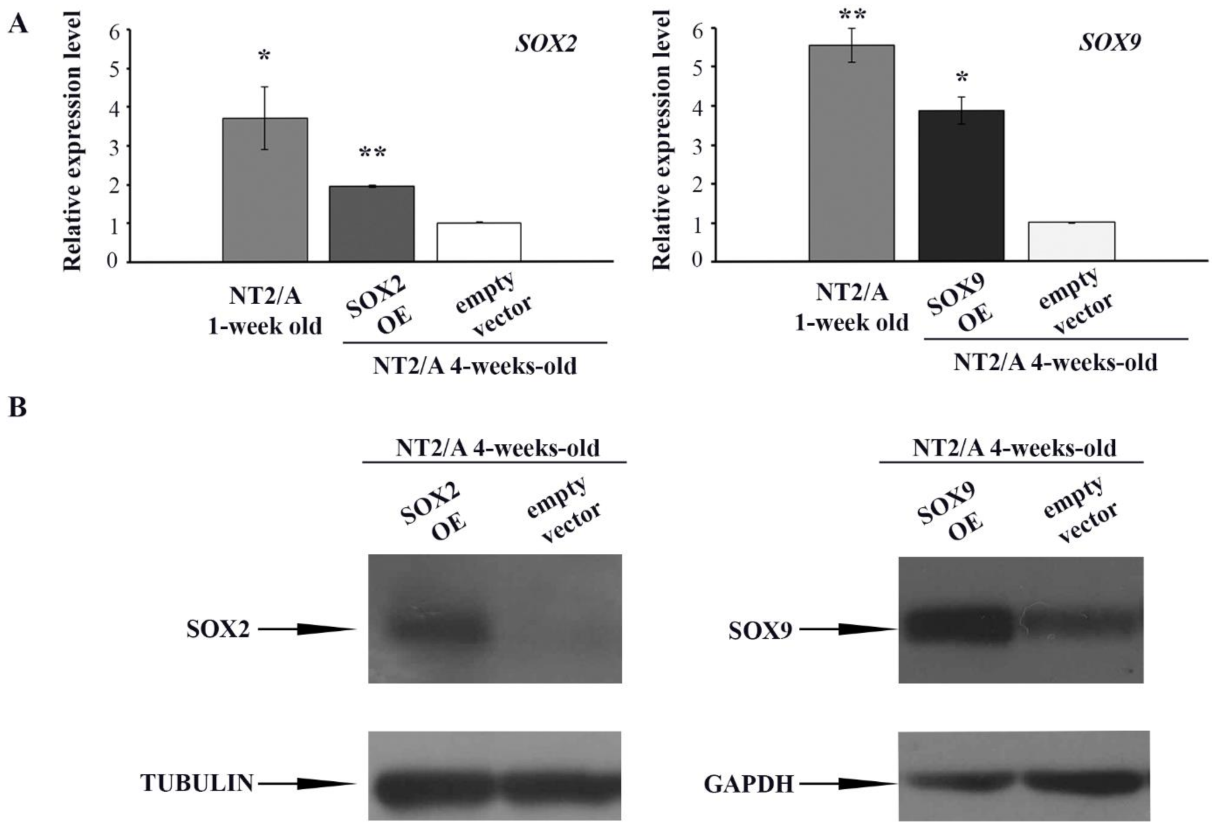

Figure S4-The analysis of migratory-capacity of 4-weeks-old NT2/A transduced with SOX2 expression vector by scratch wound healing assay.

Representative phase contrast images of wound closure experiments. The migratory capability of 4-weeks-old NT2/A transduced with *SOX2* expression vector or empty vector (control) was observed over a 36h time window. Scale bar: 100 µm. Statistical analysis of the percentage of wound closure was obtained from three independent experiments. Data represent means ± SEM, *p*-values were calculated using Student'st-test, * $p \le 0.05$, ** $p \le 0.001$.

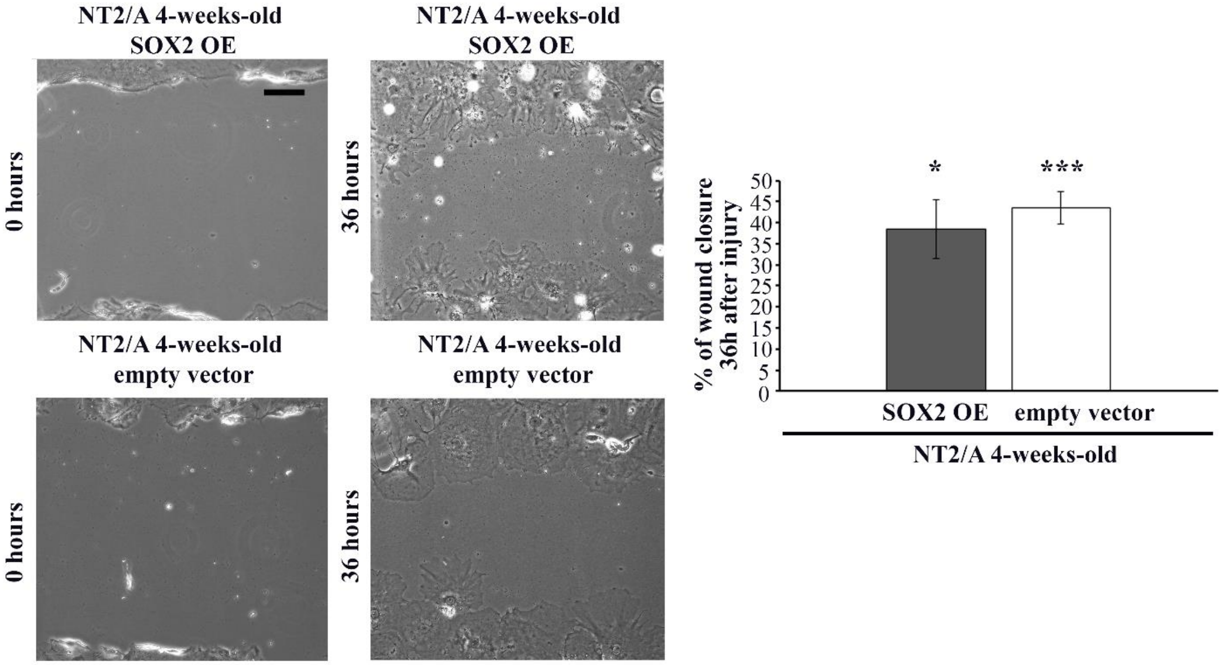

NT2/A 4-weeks-old
SOX2 OE
0 hours
36 hours
NT2/A 4-weeks-old
empty vector
% of wound closure
36h after injury
50
45
40
35
30
25
20
15
10
5
0
*
***
SOX2 OE
empty vector
NT2/A 4-weeks-old
